# In Silico Analysis of Honey Bee Peptides as Potential Inhibitors of Capripoxvirus DNA-Directed RNA Polymerase

**DOI:** 10.3390/ani13142281

**Published:** 2023-07-12

**Authors:** Ghulam Mustafa, Hafiza Salaha Mahrosh, Mahwish Salman, Muhammad Ali, Rawaba Arif, Sibtain Ahmed, Hossam Ebaid

**Affiliations:** 1 Department of Biochemistry, Government College University Faisalabad, Faisalabad 38060, Pakistan; 2 Department of Biochemistry, University of Agriculture Faisalabad, Faisalabad 38040, Pakistan; mahroshhafiz@gmail.com (H.S.M.);; 3 Department of Biochemistry, University of Jhang, Jhang 35200, Pakistan; 4 Scripps Institution of Oceanography, University of California San Diego, 9500 Gilman Drive, La Jolla, CA 92093, USA; sibtain@bzu.edu.pk; 5 Department of Biochemistry, Bahauddin Zakariya University, Multan 60800, Pakistan; 6 Department of Zoology, College of Science, King Saud University, P.O. Box 2455, Riyadh 11451, Saudi Arabia

**Keywords:** bee peptides, Capripoxvirus, goatpox, lumpy skin disease, sheeppox, molecular technologies

## Abstract

**Simple Summary:**

The viruses of the Capripoxvirus genus (i.e., sheeppox, goatpox, and lumpy skin disease viruses) pose significant financial threats to the livestock industry, causing decreased animal product output. The current study was aimed to determine the evolutionary relationships of Capripoxvirus with other Poxviridae family members through phylogenetic analysis and assess the antiviral potential of honey bee peptides against SPPV, GTPV, and LSDV. Protein–protein docking experiments were conducted, focusing on the interactions between honey bee peptides and the DNA-directed RNA polymerase of these viruses. Among the five peptides tested, mellitin and secapin-1 displayed the most favorable results, with the lowest binding scores and stable complexes. Molecular dynamics simulation further confirmed the strong connection between the protein DNA-dependent RNA polymerase and the melittin peptide, suggesting stable binding. These findings demonstrate the potential of bee peptides, particularly mellitin and secapin-1, as effective antimicrobial agents against SPPV, GTPV, and LSDV, offering a promising avenue for future research and development of antiviral therapies.

**Abstract:**

The genus Capripoxvirus belongs to the Poxviridae family. The sheeppox, goatpox, and lumpy skin disease viruses are three species of this genus with 96% identity in their genomes. These are financially devastating viral infections among cattle, which cause a reduction in animal products and lead to a loss in livestock industries. In the current study, the phylogenetic analysis was carried out to reveal the evolutionary relationships of Capripoxvirus species (i.e., sheeppox virus (SPPV), goatpox virus (GTPV), and lumpy skin disease virus (LSDV)) with other viruses from the Poxviridae family with >96% query coverage to find the similarity index among all members. The three viruses (i.e., SPPV, GTPV, and LSDV) joined the clade of Capripoxvirus of the Poxviridae family in the phylogenetic tree and exhibited close evolutionary relationships. The multiple sequence alignment using ClustalOmega revealed significant variations in the protein sequences of the DNA-dependent RNA polymerase of SPPV, GTPV, and LSDV. The three-dimensional structures of five selected bee peptides and DNA-directed RNA polymerase of SPPV, GTPV, and LSDV were predicted using trRosetta and I-TASSER and used for molecular docking and simulation studies. The protein–protein docking was carried out using HADDOCK server to explore the antiviral activity of peptides as honey bee proteins against SPPV, GTPV, and LSDV. In total, five peptides were docked to DNA-directed RNA polymerase of these viruses. The peptides mellitin and secapin-1 displayed the lowest binding scores (−106.9 +/− 7.2 kcal/mol and −101.4 +/− 11.3 kcal/mol, respectively) and the best patterns with stable complexes. The molecular dynamics simulation indicated that the complex of protein DNA-dependent RNA polymerase and the peptide melittin stayed firmly connected and the peptide binding to the receptor protein was stable. The findings of this study provide the evidence of bee peptides as potent antimicrobial agents against sheeppox, goatpox, and lumpy skin disease viruses with no complexity.

## 1. Introduction

Capripoxvirus (CaPV) is a dsDNA virus belonging to the Poxviridae family. The family is mainly comprised of the goatpox virus (GTPV), sheeppox virus (SPPV), and lumpy skin disease virus (LSDV), which cause goat pox (GTP), sheep pox (SPP), and lumpy skin disease (LSD), respectively, in ruminants [1]. The Capripoxvirus infections have a negative impact on the livelihood of poor farming communities in endemic regions. The conflicted regions will continue as a source of infection until the proper immunization of goats and cattle. Additionally, eliminating all sick and in-contact animals is neither a cheap nor a practical disease control method in any of those nations where LSD, SPP, and GTP are now endemic. SPP and GTP historically had a larger global circulation than LSD. According to the World Animal Health Information Database (WAHID) of World Organization for Animal Health (OIE), the SPP and GTP incidents are most common in the Middle East, Central Asia, and Eastern and Western Asia. The illnesses are particularly widespread in Turkey, where four outbreaks occurred between 2013 and 2015, and other outbreaks were also reported in Bulgaria and Greece [2].

The World Organization for Animal Health has designated Capripoxvirus infections as a notifiable transboundary animal diseases due to their severe economic impact and the ability of outbreaks to quickly spread across national borders [3]. SPPV and GTPV normally infect sheep and goats, respectively, whereas LSDV can infect cattle, buffaloes, and other wild ruminants. Fever, lymphadenopathy, oedema leading to lameness, and characteristic nodular skin lesions are the clinical signs of Capripoxvirus infection [3]. The chordopoxviriniae in the Poxviridae family with three viruses has a severe effect on sheep, goats, and cattle in Africa, Asia, and most recently, Eastern Europe. Sheep, goats, and cattle are affected by the lumpy skin disease virus (LSDV), goatpox virus (GTPV), and sheeppox virus (SPPV), respectively [4].

The CaPV genome is a 150 kb long, linear, and double-stranded DNA molecule that encodes between 147 and 156 open reading frames. Capripoxvirus genomes are highly similar to one another, with approximately 96–98% sequence identity across their whole genome lengths [5,6].

Sheeppox and goatpox can be spread via direct or indirect contact between infected animals or contamination by bedding [4]. The CaPVs are known to persist in scabs or lesions on the surface of the skin. The shedding of dried scabs containing viruses is the most common cause of the spreading of the virus. Approximately, one-third of the infected animals become viremic with no significant symptoms and skin lesions. These viremic animals are most capable of the rapid spread of viral infections through arthropod vectors, making efforts for the eradication of the infection harder [7].

SPPV and GTPV, which are highly infectious, can result in extremely high morbidity (70–90%) and mortality (up to 50%). Young animals exhibit more severe infection, and high fatality rates in lambs may reach up to 100% [8]. Although the virulence of various CaPVs can differ, the severity of the clinical illness is frequently influenced by the host’s species, breed, age, immunological condition, and stage of development. In the past, CaPVs were thought to be host specific. Although some viruses can affect both species, SPPV and GTPV typically display more critical infections in sheep or goats. Unexpectedly, a recent investigation in Ethiopia has revealed that GTPV was solely to blame for all outbreaks that were seen in sheep and goats throughout the study. Moreover, different Capripoxvirus outbreaks in developing countries are rarely reported due to the lack of global data repositories, resulting in an underreporting of the disease’s global burden [9].

The family Poxviridae has been classified into two subfamilies, i.e., chordopoxvirinae that infects vertebrates (e.g., birds, reptiles, mammals, and fish) and Entomopoxvirinae that infects invertebrates, which includes different orders of insects [10]. Poxviridae viruses are among the largest and most complicated viruses that replicate and assemble completely within the cytoplasm and are completely independent of the nucleus of their hosts [11]. The enveloped, pleomorphic, roughly brick-shaped or oval poxviral virions are 220–380 nm long and 140–300 nm wide, and are made up of copies of approximately 80–90 distinct viral proteins. Mature poxvirus virions are defined as having a complicated structure and lack the helical or icosahedral capsid geometries found in most of the viruses. The poxvirus genome is a double-stranded linear DNA molecule ranging in length from 127 to 365 kilobase pairs that encodes for 130 to more than 300 genes. Viral entry into the host cell is mediated via interactions between the viral capsid and receptors on infected cells. Followed by entry, the viral genome is released into the cytoplasm where it serves both as the mRNA and template, and also serves as a replication strand [12]. In cytoplasm, the virus encodes multi-subunit DNA-dependent RNA polymerase, which is responsible for an early gene expression. The complete enzyme complex consists of viral core protein E11, transcription factor VETF, and mRNA processing factors VTF/CE that carry out an early transcription [13]. Therefore, RNA polymerases (RNAPs) are transcriptional engines and a critical target for the regulation of gene expression in health and illness [14].

In 1929, LSDV was identified for the first time in Zambia; later, it was reported in several countries. Since 2015, it has expanded to Russia, Azerbaijan, Armenia, Greece, Bulgaria, Albania, Kosovo, Serbia, Montenegro, and other countries. The morbidity rate varies between 5% and 45% but sometimes reaches 100%, depending on the severity of the disease outbreak, greatly influenced by animal immunity, breed, age, and production time [15]. The morbidity and mortality rates of 8.7% and 0.4% were recorded in Greece [16] and 12.3% and 6.4%, in Turkey disease outbreaks [17]. In the past two decades, Capripoxvirus infections have been spread rapidly across the Middle East, reaching Russia, and, recently, the Asian subcontinent [18].

Capripoxvirus infections have resulted in major financial losses in the affected countries. Due to high fever and secondary mastitis, this infection affects milk yield significantly (from 10% to 85%). The additional consequences of infection include damaged skin, a decrease in beef cattle growth rate, either temporary or permanent infertility, miscarriage, treatment and vaccine expenditures, and the death of a plagued animal [15,17].

The transcription of viral DNA into RNA is catalyzed by DNA-dependent RNA polymerase using four ribonucleoside triphosphates as substrates. The enzyme is responsible for the transcription of viral genes at early, intermediate, and late stages. The enzyme is also associated with the early transcription factor (ETF) that allows the transcription of early viral genes. Late and probably intermediate transcription also require an RNA polymerase that is newly synthesized. With some exceptions because of insertions of lineage-specific domains, the active multi-subunit of DNA-directed RNA polymerase is structurally conserved among all domains of life [19]. DNA-directed RNA polymerase is therefore considered as a potential target to inhibit the viral transcription machinery and consequently suppress goatpox, sheeppox, and lumpy skin disease virus. In this study, we have therefore targeted the DNA-directed RNA polymerase of GTPV, SPPV, and LSDV by bee peptides to explore the binding pattern between bee peptides and Capripoxvirus DNA-directed RNA polymerase.

Bee venom contains a variety of diverse biological active compounds with their potential therapeutic roles in a variety of clinical aspects. The honey bee venom has been reported with a variety of antimicrobial compounds such as melittin, secapin, apamin, and mastoparan [20]. Honey bee venom contains several natural compounds such as enzymes, phytochemicals, and bioactive peptides that have potential anti-inflammatory, antiviral, and anticancer potential. Apitherapy is a type of complementary treatment that makes use of honey bee products such as honey, pollen, propolis, royal jelly, and, most importantly, bee venom (BV) [21]. Melittin, apamin, adolapin, phospholipase A2, hyaluronidase, and secapin are among the most reported honey bee venom peptides with potential applications. Melittin has been reported with potential antiviral activity against envelope and non-envelope viral strains. Phospholipase A2 displayed the blockage of viral replication machinery that inhibits the viral replication [22]. Honey bees are eusocial insects that use special defense mechanisms such as RNA Interference (RNAi), sequence-specific RNAi, and non-sequence specific dsRNA triggered pathways to respond to viral infections [23]. The aim of this research was to explore five honey bee peptides as potent inhibitors of emerging viral infections in domestic animals.

## 2. Materials and Methods

### 2.1. Selection and Retrieval of Ligand and Receptor Proteins

The amino acid sequences of Capripoxvirus DNA-directed RNA polymerase of SPPV (UniProt ID: P19749), GTPV (UniProt ID: V5KZN2), and LSDV (UniProt ID: Q8JTZ9) were retrieved from the Uniprot Database in FASTA format [24].

The amino acid sequences of proteins such as honey bee (of the Apidae family) proteins were retrieved in FASTA format from UniProt Database. Melittin (UniProt ID: P01501), apamin (UniProt ID: P01500), secapin-1 (UniProt ID: C0HLU0), cuckoo bee protein melectin (UniProt ID: P86170), and Japanese carpenter bee peptide antimicrobial peptide Xac-2 (UniProt ID: C0HKQ6) were selected as ligands.

### 2.2. Phylogenetic Analysis

Along with three Capripoxvirus species (i.e., SPPV, GTPV, LSDV), 19 similar protein sequences with query coverage more than 96% were collected through BLASTp [25]. The sequences were aligned by ClustalX and exported to MEGA format in MEGA7 program [26]. MEGA7 was used to reconstruct a Neighbor-Joining (NJ) phylogenetic tree with 100 bootstrap repetitions [27].

### 2.3. Homology Modeling, Refinement, and Validation

The multiple sequence alignment of selected Capripoxvirus proteins was performed by ClustalOmega provided by European Molecular Biology Laboratory-European Bioinformatics Institute (EMBL-EBI) [28] to check the similarities and mutations among selected proteins of the three viral strains. Chimera 1.16 [29] was used for the 3D alignment of SPPV, GTPV, and LSDV to highlight the aligned regions. The 3D structures of ligand peptides were predicted by trRosetta [30] and I-TASSER [31], refined by the GalaxyRefine server [32], and evaluated by a Ramachandran plot analysis [33] and Verify 3D [34].

### 2.4. Protein–Peptide Docking

The molecular docking was performed between the selected DNA-directed RNA polymerases of SPPV, GTPV, and LSDV and bee peptides. The SPPIDER (an online server) was used to predict the active site residues of each viral protein [35]. The molecular docking was carried out by the HADDOCK server [36] to explore specific protein–peptide interactions between selected peptides and the DNA-directed RNA polymerase of three viral variants. The educational version of the PyMOL Molecular Graphics System was used to predict and draw the interactions between the active residues of selected peptides and proteins [37]. Later, these docked complexes were further validated by PDBsum to display the intercomplex interactions [38].

### 2.5. Molecular Dynamics Simulation

The protein–peptide complex was preprocessed using a Preparation Wizard of Maestro, which included complex optimization and minimization. All systems were prepared using the System Builder tool. Transferable Intermolecular Interaction Potential 3 Points (TIP3P), a solvent model with an orthorhombic box, was chosen. In the simulation, the OPLS 2005 force field was used [39]. To make the model neutral, counter ions were introduced. To mimic the physiological conditions, 0.15 m sodium chloride (NaCl) was added. The NPT ensemble with 300 K temperature and 1 atm pressure was chosen for the entire simulation. The models were relaxed before the simulation. The trajectories were saved for examination after every 100 ps, and the simulation’s stability was verified by comparing the protein and peptide’s root mean square deviation (RMSD) and root mean square fluctuation (RMSF) over time.

## 3. Results and Discussion

### 3.1. Phylogeny of CaPVs

**Figure 1:**
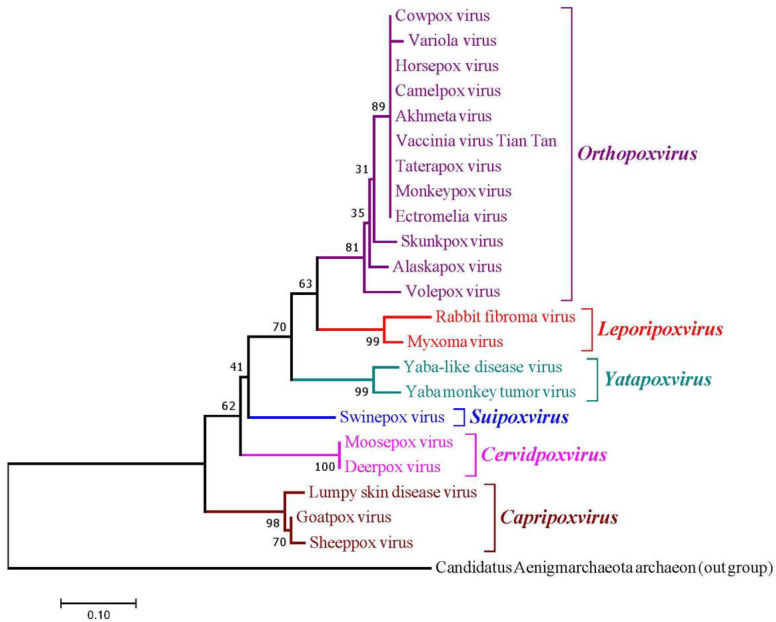
Phylogenetic tree of Capripoxviruses (CaPVs).

A phylogenetic tree of DNA-dependent RNA polymerases among Capripoxviruses (SPPV, GTPV, and LSDV) and the 19 most similar viruses was reconstructed to predict their evolutionary relationships (Figure 1). The Neighbor-Joining (NJ) method [40] was employed with the bootstrap test (100 replicates). The evolutionary distances were calculated using the Poisson correction technique [41]. The phylogenetic tree was drawn to scale (0.10) to show the number of differences between sequences. The scale of 0.10 means 10% differences between two sequences.

The phylogenetic analysis is an efficient technique to determine the evolutionary relationships among different species using their nucleotide and protein sequences [27]. In this study, the phylogram settled all viral strains into six clades (i.e., *Orthopoxvirus*, *Leporipoxvirus*, *Yatapoxvirus*, *Suipoxvirus*, *Cervidpoxvirus*, *Capripoxvirus*) based on their similarity index. The three viruses (i.e., sheeppox virus, goatpox virus, and lumpy skin disease virus) appeared in the clade of *Capripoxvirus* of the Poxviridae family and showed close evolutionary relationships. The clade *Cervidpoxvirus* included two members (i.e., *Moosepox virus* and *Deerpox virus*). *Suipoxvirus* has only one member (i.e., swinepoxvirus), which displays distant evolutionary relationships with the viruses of other clades. The clade *Yatapoxvirus* has two members that were found to be related to monkey pox infection (*Yaba-like disease virus, Monkeypox virus*). *Rabbit fibroma virus* and *myxoma virus* from the *Leporipoxvirus* clade have been reported as causative agents for localized cutaneous fibroma in rabbits. The largest clade, *Orthopoxvirus,* has nine members and all belong to *Poxviruses*. These viruses are associated with multiple infections such as cowpox, camelpox, monkeypox, and horsepox, which also infect humans. The phylogenetic analysis of all viruses has one thing in common that they all belong to *Poxviridae*, which is the key element that provides evidence of similarity at their genomic level. In a study, Sumana et al. [42] reconstructed a phylogenetic tree of 28 isolates from 25 SPPV and GTPV outbreaks based on P32 gene/protein sequence. They revealed that LSDV and SPPV showed a closer evolutionary relationship compared to GTPV. Similarly, in another phylogenetic study [43], it was revealed that SPPV, GTPV, and LSDV isolates from Iran clustered with SPPV, G GTPV PPV, and LSDV, which were retrieved from GenBank with 99%, 98–99%, and 99–100% sequence identities in the chemokine receptor gene, respectively. The phylogeny can be used to correctly diagnose the endemic viral strains, which will guide the veterinary managers for choosing a homologous vaccine. In a study, Saidi [44] generated a phylogenetic tree of SPPV, GTPV, and LSDV and revealed that all viruses clustered in their respective clades and hence were distinguished from each other.

### 3.2. Homology Modeling

**Figure 2:**
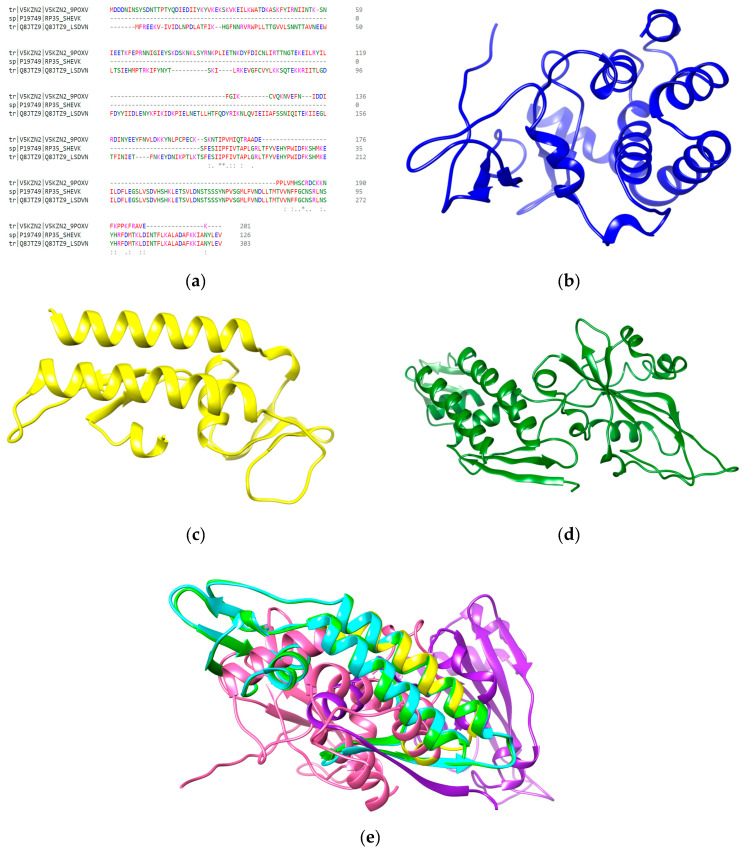
(**a**) Multiple sequence alignment of DNA-dependent RNA polymerase of selected Capripoxviruses (i.e., goatpox virus (V5KZN2), sheeppox virus (P19749), and lumpy skin disease virus (Q8JTZ9)), where asterisks (*) are indicating positions where a single, fully conserved residue is found, colon (:) is indicating conservation among residues with strongly similar properties, and the period (.) is indicating conservation among residues of weakly similar properties. Predicted 3D models of DNA-directed RNA polymerase of (**b**) goatpox virus, (**c**) sheeppox virus, (**d**) lumpy skin disease virus. (**e**) Superimposition of 3D structures of viral proteins.

The multiple sequence alignment of DNA-dependent RNA polymerase of SPPV, GTPV, and LSDV indicated substantial variations among their protein sequences, which make this protein a perfect target to stop the transcriptional machinery (Figure 2a). Similar sequences are shown in the same color. Due to the non-availability of suitable templates, the most reported tools (trRosetta and I-TASSER) were used for the homology modeling of bee peptides as well as Capripoxvirus receptor proteins. The amino acid sequences of Capripoxvirus DNA-directed RNA polymerase of GTPV (Figure 2b), SPPV (Figure 2c), and LSDV (Figure 2d) were subjected to I-TASSER for 3D modeling. The I-TASSER uses a multiple threading approach to predict the protein structure based on PDB structural templates. The best model of each protein was selected for further analyses on the basis of their RMSD values, GDT-HA, and C-scores. The 3D structures of DNA-dependent RNA polymerase of SPPV, GTPV, and LSDV were aligned using a UCSF Chimera to find similar amino acid residues among them (Figure 2e). The superimposition of 3D structures of viral proteins show the regions of homology obtained by the alignment of the Capripoxviruses, LSDV (purple), GTPV (Hot pink), and SPPV (blue) and the aligned regions of LSDV (cyan), SPPV (green), and GTPV (yellow) (Figure 2e).

**Figure 3:**
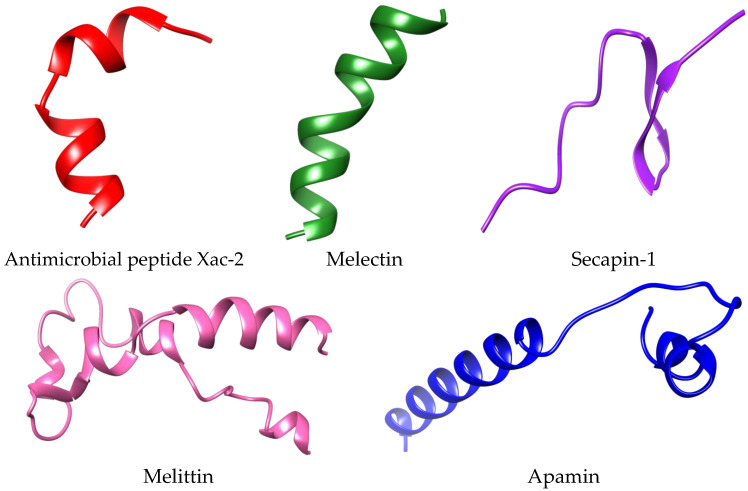
3D models of bee peptides predicted using trRosetta server.

The 3D structures of five bee peptides including melittin (amino acid sequence: MKFLVNVALVFMVVYISYIYAAPEPEPAPEPEAEADAEADPEAGIGAVLKVLTTGLPALISWIKRKRQQR), apamin (amino acid sequence: MISMLRCIYLFLSVILITSYFVTPVMPCNCKAPETALCARRCQQHG), secapin-1 (amino acid sequence: YIINVPPRCPPGSKFVKNKCRVIVP), melectin (amino acid sequence: GFLSILKKVLPKVMAHMK), and antimicrobial peptide Xac-2 (amino acid sequence: GFVALLKKLPLILKHLP) were constructed using an online server trRosetta (Figure 3). The trRosetta is a deep network that predicts the inter-residue geometry to guide structure prediction based on direct energy minimization, as implemented in the ros framework [30].

**Figure 4:**
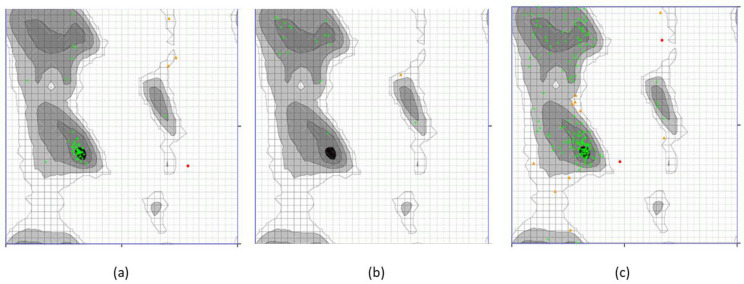
Ramachandran plot analysis of the best predicted models. (**a**) Melittin, (**b**) secapin-1, (**c**) goatpox virus.

The Galaxy refine server was employed for the refinement of the best predicted models of each protein, which enhanced the Rama-favored regions from the initial 60% to 90% after adjusting the local infrastructure of the predicted models. Furthermore, the stability and accuracy of the predicted models were evaluated by Ramachandran plot analysis. About 85% of the amino acids of the predicted models fell in the Rama-favored regions, which signifies the accuracy of the predicted models. The top models with the best HADDOCK scores, with melittin representing 93.22%, secapin-1 showing 94.44%, and goatpox virus showing 93.55% of residues in the Ramachandran plot favored regions (Figure 4). The green color shows highly preferred observations, orange and black grids represent the preferred observations.

### 3.3. Protein–Peptide Docking

Docking predicts the interactions and binding patterns between different peptides/ligands and receptor proteins. Due to the limited data available on Capripoxvirus, the 3D structure of each peptide and viral protein was predicted and evaluated by notable web tools. SPPIDER online web server was employed to predict the interaction sites of the sheeppox virus, goatpox virus, and lumpy skin disease virus. The five bee peptides were docked to DNA-directed RNA polymerase of selected Capripoxviruses using HADDOCK server. The complexes with the lowest binding energy and the best binding patterns were considered as suitable ones.

DNA-directed RNA polymerase of Capripoxviruses catalyzes the viral DNA transcription into RNA and also responsible for the transcription of early, intermediate, and late genes. Therefore, the DNA-directed RNA polymerase of SPPV, GTPV, and LSDV, was selected in this study as a target receptor protein to stop the CapV infection. The bee peptides were used as ligand molecules and docked counter to the DNA-directed RNA polymerase of these viruses. A total pf five peptides (melittin, apamin, melectin, Xal-2, and secapin-1) were docked counter to the three receptor proteins of CaPV. The protein–peptide complexes with the lowest binding energy were considered as the best ones and selected for further analysis. In the current study, melittin and secapin-1 showed the lowest binding energy with strong interactions with the goatpox virus (Table 1).

Capripoxvirus belongs to the Poxviridae family, mainly composed of the goatpox virus, sheeppox virus, and lumpy skin disease virus. These viruses are characterized among the largest viruses with closely identical genomes (96%) at their nucleotide levels. The virulence of CaPV infection may vary with SPPV and GTPV as the most virulent species (90%) compared to LSDV (45%) in different cases, which depends on the age and immune system of the infected animals [45]. All these infections have a great impact on the economy of a country due to losses in meat, wool, milk, and cashmere production [46]. The transmission of SPPV, GTPV, and LSDV is poorly understood, particularly in endemic areas. Previous research indicated that SPPV and GTPV spread via aerosol and direct contact with mechanical transmission by insect vectors, which play only a minor role. The LSDV, on the other hand, is considered as primarily transmitted by blood-feeding insects [2,46,47]. Animal movements, the gathering of animals from different herds in close contact, and the introduction of new animals (without quarantine) into naive herds have all been identified as important risk factors for SP, GP, and LSD [46].

Protein–protein/peptide interactions play a crucial role in exploring the structural and functional patterns among different complexes. Molecular docking is an emerging technique that mediates the understanding of the underlying complex interactions among a variety of biomolecules using computers [48,49].

### 3.4. Interactions between Bee Peptides and Capripoxvirus DNA-Dependent RNA Polymerase

**Figure 5:**
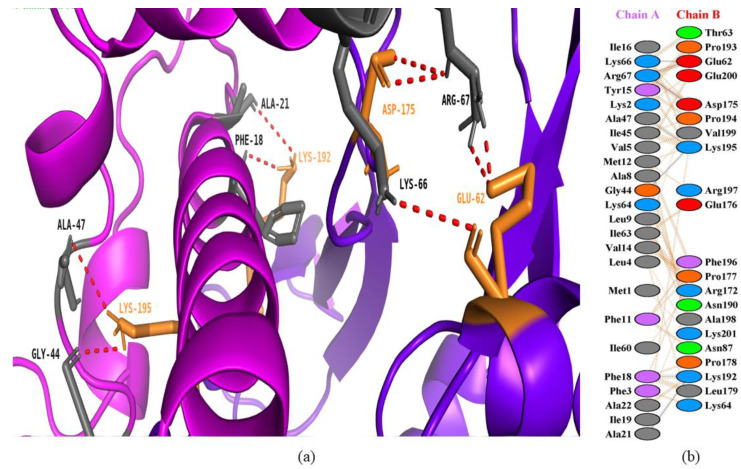
Protein–peptide interactions between honey bee venom protein (melittin) and goatpox viral DNA-dependent RNA polymerase. (**a**) Melittin is represented in a magenta color with gray interacting residues, and GTPV is shown in purple-blue color with orange interacting residues. (**b**) Interacting residues between melittin and GTPV.

In the current study, melittin, the honey bee venom peptide, with a HADDOCK score of −106.9 +/− 7.2 kcal/mol, showed a good binding pattern with the predicted active site residues of the goatpox viral DNA-dependent RNA polymerase (Figure 5). Melittin is an *Apis mellifera* L. (honey bee) venom peptide with strong hemolytic and antimicrobial activity. This honey bee venom is comprised of a wide range of complex therapeutic compounds and peptides that enable bees to defend their hives against predators and external threats [50]. The DNA-dependent RNA polymerase of CaPV regulates the viral transcriptional factory, responsible for early, intermediate, and late gene transcription. This shows that the targeting of DNA-dependent RNA polymerase is the leading way towards the inhibition of viral replication.

The World Organization for Animal Health (OIE) has characterized goatpox and sheeppox infections as notifiable ones due to a high rate of mortality. Internationally, CaPV infections bring a notable reduction in animals and their product trade, which brings disasters to a particular nation due to economic losses. SPPV and GTPV are more virulent with 90% infection with mild to severe clinical symptoms in animals.

**Figure 6:**
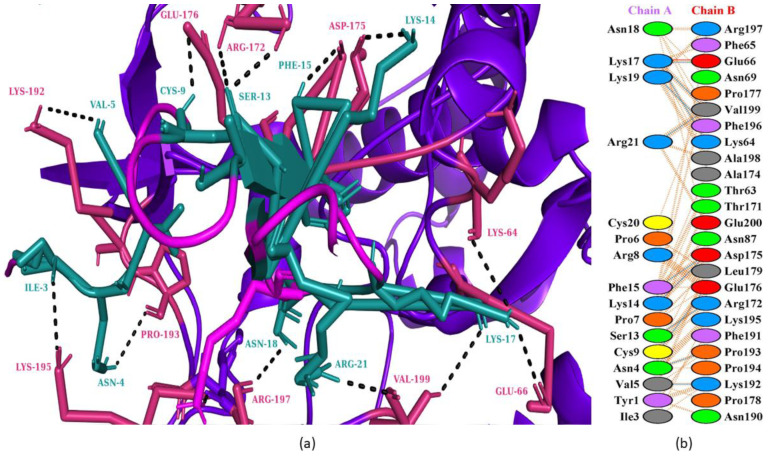
Protein–peptide interactions between honey bee protein (secapin-1) and goatpox viral DNA-dependent RNA polymerase. (**a**) Secapin-1 is represented in a magenta color with deep teal interacting residues, and GTPV is shown in purple-blue color with warm pink interacting residues. (**b**) Interacting residues between secapin-1 and GTPV.

Similarly, in the study, secapin-1 with a HADDOCK score of −101.4 +/− 11.3 kcal/mol also showed strong interactions with the predicted active residues of DNA-dependent RNA polymerase of the goatpox virus (Figure 6). Secapin-1 is an *Apis mellifera* (honey bee) serine protease with reported antimicrobial and antifibrinolytic activities. Secapin-1 displayed a wide range of antibacterial and antifungal activities against different bacterial and fungal strains.

### 3.5. Molecular Dynamics (MD) Simulation

For 100 nanoseconds, Desmond, a software from Schrödinger LLC, NY, USA (version 2019.4), was used to model the molecular dynamics [51]. The earliest phase of the receptor and peptide complex for molecular dynamics simulation was the docking experiments. Molecular docking studies can predict the ligand binding state in static situations. Docking is useful because it provides a static view of a molecule’s binding pose at the active site of a receptor [52]. By integrating Newton’s classical equation of motion, MD simulations typically compute atom movements over time. Simulations were used to predict the ligand binding status in the physiological environment [53,54].

**Figure 7:**
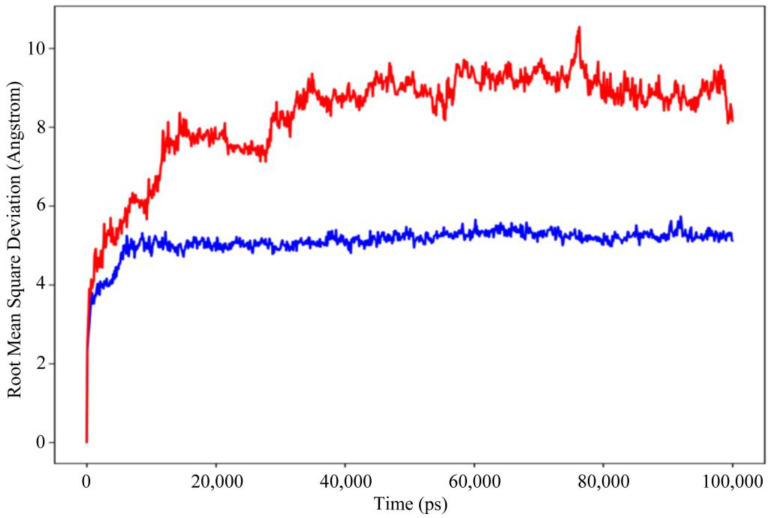
Root mean square deviation (RMSD) of the receptor protein and peptide with time. The left *y*-axis shows the variation of RMSD through time.

Figure 7 depicts the evolution of the RMSD values for the bases of DNA-dependent RNA polymerase and melittin complex over time. The blue color indicates the receptor protein and the red shows the peptide. The plot shows that the complex reaches stability at 20 ns. After that, for the length of the molecular dynamic simulation, the fluctuations in RMSD values for the target remained within 1.0 Å, which is absolutely acceptable. The peptide was fit to the receptor RMSD values and fluctuated within 2.0 Å after they were equilibrated. These findings indicate that the peptide stayed firmly connected to the receptor binding site throughout the simulation period.

**Figure 8:**
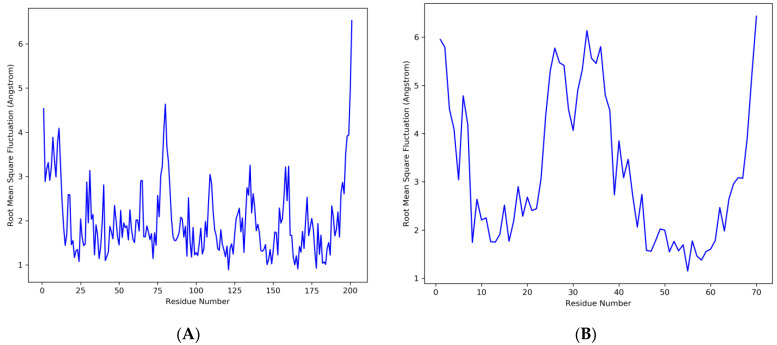
Residue wise root mean square fluctuation (RMSF) showing the most fluctuated regions in the DNA-directed RNA polymerase as receptor protein and the ligand. (**A**) Receptor protein, (**B**) peptide as a ligand.

On the RMSF graph, the peaks represent the portions of the residues that fluctuated the most during the simulation (Figure 8). Tails typically change more than any other part of the protein. Low RMSF values of the binding site residues indicate that the ligand binding to the protein is stable.

**Figure 9:**
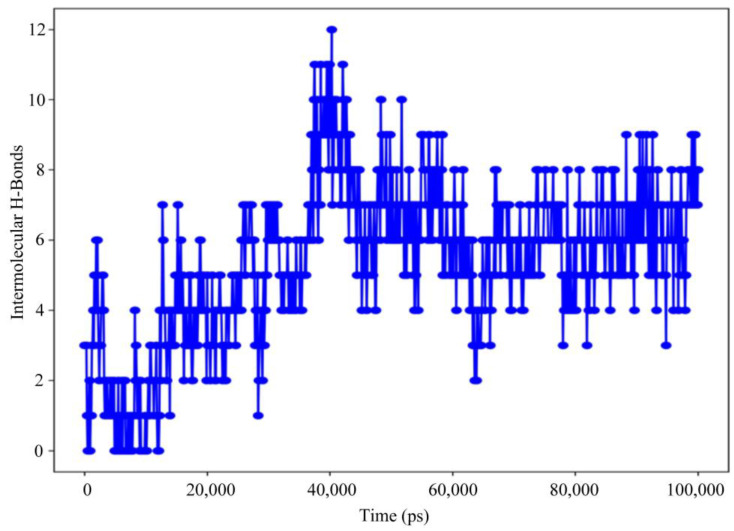
Protein–peptide contact hydrogen bonds.

The receptor protein interactions with the peptide can be detected throughout the simulation. As seen in Figure 9, the majority of the significant protein–peptide interactions discovered by molecular dynamics are hydrogen bonds. A timeline depicts the interactions and contacts (H-bonds) described.

Lee et al. [55] expressed recombinant mature AcSecapin-1 peptide that binds to the bacterial and fungal cellular surface. The outcomes of their study revealed the antibacterial, antifungal, and antifibrinolytic activity of AcSecapin-1.

Muzammal et al. [56] screened nine venom proteins and docked to normal and mutated spike proteins of Ebola virus. Computational approaches including homology modeling and protein–protein docking were carried out to understand the binding modes of venom proteins to Ebola protein. The study indicated a strong antiviral activity of melittin and phospholipase A2 peptides found in the honey bee venom.

Similarly, Burranboina et al. [57] investigated 19 phytochemicals from the leaf extract of *Leucas aspera* against Capripoxvirus p32 and RNA polymerase. These phytochemicals were docked along with FDA approved drugs against the Capripoxvirus receptor proteins. Their study displayed imidazole, 2,6-dimethylbenzaldehyde carbamoylhydrazone, n-hexylmethanesulfonamide, N’-[(E)-(4-bromophenyl)methylidene]-4-methylbenzohydrazide, 4-(2-amino-1-methylethyl), and methyl-N-hydroxybenzenecarboximidate as accepted potential drug candidates after molecular docking and pharmacokinetics analysis. In another study, Pashupathi et al. [1] designed a chimeric vaccine construct of the EEV membrane glycoprotein of LSD virus, B5R goatpox virus, SPPV-ORF 117 of sheeppox virus, and common P32 protein of all three viruses. The new construct was mediated by universal T-helper agonists and several adjuvants with defined immunogenic domains. The immunogenicity and MD simulations revealed the satisfactory behavior of the final vaccine construct in stimulating humoral immunity.

Kar et al. [58] designed multi-epitope protein, which was highly conserved, non-homologous, and antigenic to bovine. The modelled vaccine subunit interacted highly with TLR4 receptor leading towards the prediction of the potential vaccine candidate against lumpy skin disease. Similarly, Enayathullah et al. [59] reported the antiviral activity of antibacterial peptides gramicidin S and melittin as therapeutic molecules for the treatment of SARS-CoV-2 infections. Both peptides tested positive for viral clearance in SARS-CoV-2 infected Vero cells after 12 h, with a maximum viral clearance after 24 h.

Pérez-Delgado et al. [60] evaluated the antibacterial activity of *Apis mellifera* venom against *E. coli*, *P. aeruginosa*, and *S. aureus*. The outcome of their study showed the minimum inhibitory concentration (MIC) of 6.88 µg/mL against *E. coli* with no satisfactory results against *P. aeruginosa*, and *S. aureus*. To summarize, venom of *A. mellifera* consists of potential bioactive molecules with leading antibacterial activity against *E. coli.*

The current study focused on the antiviral activity of honey bee peptides using computational biology approaches to filter out specific antiviral peptides supported and validated by different in silico analyses. All the reported studies discussed above indicated the importance of bee venom peptides against a variety of microbial strains, including those of bacteria, viruses, and fungi. This indicates the bioactive potential of bee venom peptides to hinder the replication of different pathogens. Therefore, in the light of previous studies regarding bee peptides, we chose DNA-directed RNA polymerase of Capripoxviruses to inhibit the viral replication to overcome the ongoing infection crisis. The outcomes of this study would help researchers to find and develop effective peptides as drug candidates against Capripoxviruses. Moreover, there is a need for different in vivo and in vitro analyses on the peptides used in this study to further confirm the results in the experimental domain.

## 4. Conclusions

Lumpy skin disease is a catastrophic bovine disease that has gained widespread attention due to its swift propagation around the world with great livestock morbidity. In the current study, the key elements were identified to target the transcriptional machinery of Capripoxviruses to stop their early to late gene transcription using different natural peptides. It has been revealed that melittin and secapin-1 from honey bee venom have much more potential to inhibit viral replication. The molecular dynamics simulation study proved that the peptide melittin was stable in binding to the receptor protein throughout the simulation period and stayed firmly connected. Further research is required to support and explore more about this aspect in future.

## Figures and Tables

**Table 1:** HADDOCK scores and sources of selected bee peptides docked to Capripoxviruses.

Peptide	Source	DNA-Directed RNA Polymerase		
		Sheeppox Virus	Goatpox Virus	Lumpy Skin Disease Virus
Melittin	*Apis mellifera* (Honey bee)	−21.1 +/− 9.2	−106.9 +/− 7.2	−36.8 +/− 5.8
Apamin	*Apis mellifera* (Honey bee)	−44.3 +/− 2.7	−78.2 +/− 2.3	−25.8 +/− 12.1
Melectin	*Melecta albifrons* (Cuckoo bee)	−39.1 +/− 6.1	−86.0 +/− 7.2	12.7 +/− 4.2
Xal-2	*Xylocopa appendiculata circumvolans* (Japanese carpenter bee)	−22.0 +/− 5.0	−79.5 +/− 5.6	34.4 +/− 4.8
Secapin-1	*Apis mellifera* (Honey bee)	−48.7 +/− 3.3	−101.4 +/− 11.3	−4.5 +/− 12.8

## Data Availability

Not applicable.
